# Goal-related navigation of a neuromorphic virtual robot

**DOI:** 10.1186/1471-2202-13-S1-O3

**Published:** 2012-07-16

**Authors:** Laurence C Jayet Bray, Emily R Barker, Gareth B Ferneyhough, Roger V Hoang, Bobby D Bryant, Sergiu M Dascalu, Frederick C Harris

**Affiliations:** 1 Department of Computer Science & Engineering, University of Nevada, Reno, 89557, USA

## 

The field of biologically inspired technology has evolved to the emergence of robots that operate autonomously. Some studies have focused on developing social robots that interact with humans by following social behaviors where other research have centered their efforts on mobile robots with the ability to navigate in their well-known environment. These general-purpose autonomous robots can perform a variety of functions independently, from recognizing people or objects to navigating in a familiar room. As of yet, no humanoid robot has been capable of traveling through a new suburban environment to reproduce goal-related learning and navigational activities.

Based on experimental findings, we propose a computational model that is composed of critical interacting brain regions and utilizes fundamental learning mechanisms. It is incorporated in a sophisticated robotic system where a virtual robot navigates through a new environment, learns and recognizes visual landmarks, and consequently makes correct turning decisions to reach a reward.

The detailed brain architecture included visual, entorhinal, prefrontal and premotor cortices, as well as the hippocampus. Our microcircuitry replicated some fundamental mammalian dynamics, which were integrated in a robotic loop. This virtual robotic system was designed around a number of components unique to our NeoCortical simulator (NCS) and our Virtual NeuroRobotic (VNR) paradigm. The neural simulation was executed on a remote computing cluster and was networked to the other system components (NCSTools, Webots, Gabor filter) using our Brain Communication Server (BCS), a server developed specifically for integration with NCS.

The virtual humanoid was able to navigate through a new virtual environment and reach a reward after a sequence of turning actions. Along the way, it encountered familiar and non-familiar external cues to provide guidance and follow the correct direction. This is the first bio-inspired robot that showed high functionality during navigation while utilizing spiking cortical neurons in a real-time simulation. More importantly, it could take us a step closer to understanding memory impairments in Alzheimer’s patients.

